# Polyploid giant cancer cells induced by Docetaxel exhibit a senescence phenotype with the expression of stem cell markers in ovarian cancer cells

**DOI:** 10.1371/journal.pone.0306969

**Published:** 2024-07-11

**Authors:** Song Zhao, Lili Wang, Mingyue Ouyang, Sining Xing, Shuo Liu, Lingyan Sun, Huiying Yu

**Affiliations:** Laboratory of Basic Medicine, General Hospital of Northern Theater Command, Shenyang, China; Brigham and Women’s Hospital, UNITED STATES OF AMERICA

## Abstract

Docetaxel (Doc) plays a crucial role in clinical antineoplastic practice. However, it is continuously documented that tumors frequently develop chemoresistance and relapse, which may be related to polyploid giant cancer cells (PGCCs). The aim of this study was investigate the formation mechanism and biological behavior of PGCCs induced by Doc. Ovarian cancer cells were treated with Doc, and then the effect of Doc on cellular viability was evaluated by MTT assay and microscopic imaging analysis. The biological properties of PGCCs were further evaluated by Hoechst 33342 staining, cell cycle and DNA content assay, DNA damage response (DDR) signaling detection, β-galactosidase staining, mitochondrial membrane potential detection, and reverse transcription-quantitative polymerase chain reaction. The results indicated that Doc reduced cellular viability; however, many cells were still alive, and were giant and polyploid. Doc increased the proportion of cells stayed in the G2/M phase and reduced the number of cells. In addition, the expression of γ-H2A.X was constantly increased after Doc treatment. PGCCs showed senescence-associated β-galactosidase activity and an increase in the monomeric form of JC-1. The mRNA level of octamer-binding transcription factor 4 (*OCT4)* and krüppel-like factor 4 (*KLF4)* was significantly increased in PGCCs. Taken together, our results suggest that Doc induces G2/M cell cycle arrest, inhibits the proliferation and activates persistent DDR signaling to promote the formation of PGCCs. Importantly, PGCCs exhibit a senescence phenotype and express stem cell markers.

## Introduction

Doc is well-known for its critical role in antineoplastic practice in several types of malignancies, including ovarian cancer [1]. However, the clinical practice is limited by the development of drug resistance and cancer recurrence [2]. Taxane-based therapeutic intervention, such as Doc, could induce the formation of PGCCs [3, 4], which can inhibit tumor growth. Although most of PGCCs undergo cell death, some can survive and maintain metabolic activity [5]. PGCCs are commonly found in many advanced cancers and chemotherapy-resistant cancers [6]. Moreover, PGCCs possess stem cell-like properties and can function as blastomere-like stem cells [7]. Thus, PGCCs may play an important role in drug resistance and cancer recurrence. Targeting PGCCs may provide new opportunities for cancer therapy.

In many malignant tumors, cellular senescence is triggered in response to chemotherapy, referred to as chemotherapy-induced senescence (TIS) [8]. For a long time, TIS has been considered as contribution to the efficacy of therapy. New findings reveal that TIS plays a role in chemotherapy-induced global changes including transcriptional, epigenetic, morphological, and metabolic alterations [9], which may promote tumor relapse, metastasis, and resistance to therapy. The harmful effects of TIS have been considered. Accumulating evidence reveals that the removal of TIS cells is profit to improve the therapeutic effect [10, 11]. Because of its multiple roles, cellular senescence could serve as an important target in cancer therapy.

This work aimed to study the effects of Doc on ovarian cancer with special emphasis on the formation mechanism and biological behavior of PGCCs. We found that Doc inhibited the cellular viability; however, a lot of cells were still alive and underwent morphological change. We also revealed that Doc induced G2/M cell cycle arrest and inhibited the proliferation to promote the formation of PGCCs. PGCCs still maintained metabolic activity and seemed to possess a senescence phenotype, including flattened morphology, irreversible arrest of cell cycle progression and proliferation inhibition, activation of persistent DDR signaling, elevated lysosomal enzyme activity and loss of mitochondrial membrane potential. Importantly, we found that the expression of stem cell markers was significantly increased with the formation of PGCCs.

## Materials and methods

### Reagents

Doc was purchased from Selleckchem (Houston, TX, USA) and dissolved in dimethyl sulfoxide (DMSO; Sigma-Aldrich; St. Louis, MO, USA) to prepare a 50 mM stock. 3-(4,5-Dimethylthiazol-2-yl)-2,5-diphenyltetrazolium bromide (MTT) was purchased from Sigma-Aldrich and dissolved in phosphate buffer saline (PBS) to prepare a 5 g/L stock. Propidium iodide was purchased from Sigma-Aldrich and dissolved in PBS to prepare a 10 g/L stock. Cyanine dye JC-1 was purchased from Beijing Solarbio Science & Technology Co., Ltd. (Beijing, China) and dissolved in Roswell Park Memorial Institute (RPMI) 1640 medium (Basalmedia Technologies Co., Ltd.; Shanghai, China) to prepare a 1 g/L stock. Rabbit monoclonal antibody against γ-H2A.X (Alexa Fluor® 488 conjugate) was purchased from Cell Signaling Technology (Beverly, MA, USA). All reagents were stored at -20°C prior to use.

### Cell lines and cell culture

The human ovarian cancer cell lines SKOV3 and A2780 were obtained from iCell Bioscience Inc (Shanghai, China). Cells were maintained in the RPMI 1640 medium supplemented with 10% fetal bovine serum (FBS; VivaCell; Shanghai, China) in a humidified atmosphere of 5% CO_2_ at 37°C.

### MTT assay

The MTT assay was performed to investigate the effect of Doc on cell viability. Briefly, SKOV3 and A2780 cells were seeded into a 96-well culture plate (5×10^3^ cells/well) and treated with different concentrations of Doc (0, 0.05, 0.1, 0.5, 1, 5, 10, 25, 50, 100, 500, 1000 nM) overnight (16–18 h). 20 μL of MTT (5 g/L) was added into each well and the incubation was continued for another 4 h at 37°C. The medium was removed and formazan crystals were dissolved in 150 μL of DMSO. Cell viability was estimated by measuring the optical density (OD) level at 490 nm. The percentage of cellular viability was calculated as (%): (OD exp /OD con) ×100, where OD exp and OD con were the OD values of Doc-treated cells and control cells, respectively.

### Microscopic imaging analysis and cell counting

At 60–70% confluence, SKOV3 and A2780 cells were treated with different concentrations of Doc (200, 400, 800 nM) overnight (16–18 h), and then allowed to recover in normal medium for another three days. The morphology of cells was imaged using a microscope (Nikon, Japan) followed by processing with the NIS-Elements D 3.2 software. The diameter of cells was determined with help of the image pro plus6.0 software. After imaging, all tangible cells were harvested and counted in a hemocytometer chamber under a microscope.

### Treatment schedule for PGCCs generation

At 60–70% confluence, SKOV3 and A2780 cells were treated with Doc (800 nM) overnight (16–18 h) (i.e., the Doc group) and then allowed to recover in normal medium for another three days to form PGCCs (i.e., the PGCCs group). DMSO was added to the culture system as a vehicle control, i.e., the control group.

### Hoechst 33342 staining

SKOV3 and A2780 cells were seeded into a 24-well culture plate (ABC Biochemistry; Hong Kong, China) and treated with Doc. The cells were then rinsed once with PBS, fixed in 4% paraformaldehyde for 20 min and incubated with Hoechst 33342 (1:50) for 30 min at 37°C. The nucleus morphology of cells was imaged utilizing a Ti-S inverted fluorescence microscope (Nikon, Japan).

### Flow cytometry analysis of cell cycle and DNA content

After exposure to Doc, SKOV3 and A2780 cells (5×10^5^) were harvested, washed, and fixed with 80% ice-cold methanol and stored at -20°C for at least 12 h. The cells were then washed and incubated with propidium iodide (50 mg/L) in the dark for 30 min at room temperature. Cells were analyzed immediately using a FACS Canto® II flow cytometer (BD Biosciences; San Diego, CA, USA) followed by processing with the FlowJo 7.6 software for cell cycle and the BD FACSDiva software for DNA content.

### Flow cytometry analysis of γ-H2A.X level

After exposure to Doc, SKOV3 and A2780 cells (5×10^5^) were harvested, washed, and fixed with 4% formaldehyde for 15 min at room temperature. The cells were then washed and permeabilized with 2 mL of ice-cold 90% methanol for 2 h at -20°C. The cells were washed twice, resuspended with 100 μL of PBS containing 0.5% BSA and stained with the rabbit monoclonal antibody against γ-H2A.X (1:50) for 1 h at room temperature. The cells were then washed, resuspended in 300 μL of PBS and analyzed immediately using a FACS Canto® Ⅱ flow cytometer followed by processing with the BD FACSDiva software.

### Senescence-associated β-galactosidase assay

After exposure to Doc, the cells were stained using a senescence β-galactosidase staining kit (Cell Signaling Technology; Boston, MA, USA). Briefly, SKVO3 and A2780 cells were seeded into a 24-well culture plate and treated with Doc. The cells were washed twice with PBS and fixed with 1 × fixative solution. The cells were washed and incubated overnight at 37°C with β-galactosidase staining solution. The cells were examined under a Ti-S inverted fluorescence microscope for the development of blue color, which represents cellular senescence.

### Flow cytometry analysis of mitochondrial membrane potential (Ψm)

After exposure to Doc, SKOV3 and A2780 cells (1×10^5^) were harvested and resuspended in 1 mL of RPMI 1640 medium. JC-1 was then added at a final concentration of 2.5 mg/L, and incubated for 30 min at 37°C. Cells were analyzed immediately using a FACS Canto® Ⅱ flow cytometer followed by processing with the BD FACSDiva software. The change in Ψm in each group of cells was calculated based on the percentage of JC-1 monomers [12].

### Reverse transcription-quantitative polymerase chain reaction (RT-qPCR)

After exposure to Doc, SKOV3 and A2780 cells (5×10^5^) were harvested and total RNA was extracted using an RNAiso Plus reagent (TaKaRa Bio, Japan). According to the manufacturer’s instructions, 1 μg of RNA was used as a template for cDNA synthesis using a reverse transcription kit (TaKaRa Bio, Japan). The cDNA produced was then analyzed by quantitative polymerase chain reaction (qPCR) using a TB Green Premix Ex Taq (TaKaRa Bio) and quantified using a CFX96 Real-Time qPCR detection system (Bio-Rad Laboratories; Hercules, CA, USA). The polymerase chain reaction (PCR) thermocycling conditions were configured as follows: initial denaturation at 95˚C for 5 min; subsequent 40 cycles of 95˚C for 30 sec and 60˚C for 30 sec. The target mRNA level was analyzed using the 2^-ΔΔCt^ method [13] and all quantities were expressed as the number of folds relative to the expression of glyceraldehyde-3-phosphate dehydrogenase (GAPDH). The sequence of primers in this experiment was listed as follows:

*KLF4* forward, 5’-TGGACCCCCTCTCAGCAAT-3’

*KLF4* reverse, 5’-CAGCACTTCCTCAAGACCCAG-3’

*OCT4* forward, 5’-AAGCGATCAAGCAGCGACTAT-3’

*OCT4* reverse, 5’- GAAAGGGACCGAGGAGTACAGT-3’

*GAPDH* forward, 5’-CAACTTTGGTATCGTGGAAGGACT-3’

*GAPDH* reverse, 5’-TGATGTTCTGGAGAGCCCCG-3’.

### Statistical analysis

All bar graph results are represented as mean ± standard deviation (S.D.) of at least three independent experiments. Statistical analysis was performed with the SPSS 27.0 software. Student’s t-test was used to determine the statistical significance of the differences between two groups. Comparisons between multiple groups were analyzed using one-way analysis of variance (ANOVA) followed by LSD post-hoc test. *P*<0.05 was considered statistically significant, and *or^#^, **or^##^, and ***or^###^ denote *P*<0.05, 0.01, and 0.001, respectively, in the figures.

## Results

### Doc reduces the cellular viability

**Fig 1:**
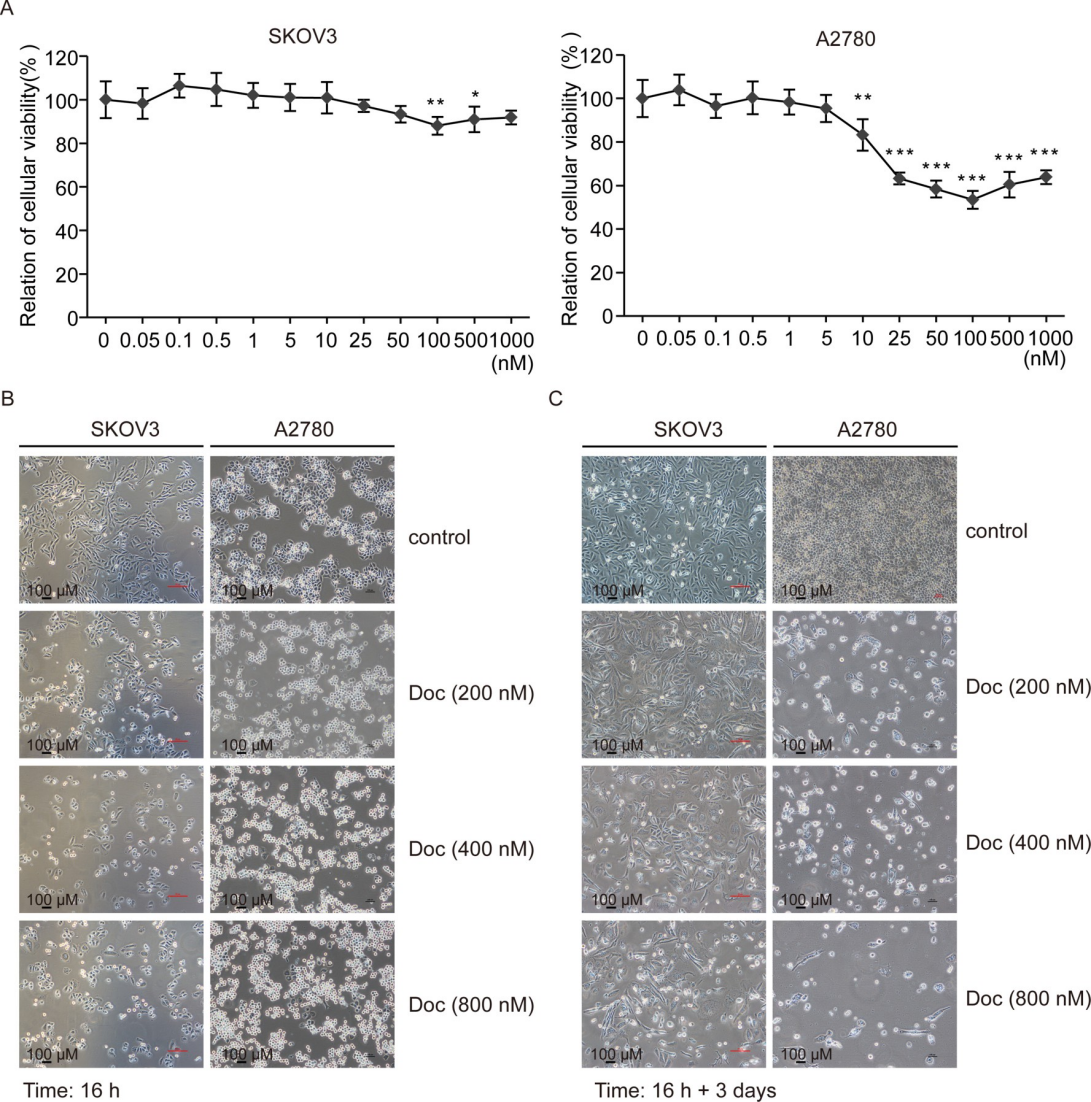
Doc reduced the cell viability. (A) SKOV3 and A2780 cells were treated with different concentrations of Doc (0, 0.05, 0.1, 0.5, 1, 5, 10, 25, 50, 100, 500, 1000 nM) overnight (16–18 h). The cell viability was determined by MTT assay (N = 5). *P* values were calculated using the student’s t-test. *P<0.05, **P<0.01, ***P<0.001 (*vs*. 0 nM). (B and C) SKOV3 and A2780 cells were treated with Doc (200, 400,-800 nM) overnight (16–18 h) (B), and then allowed to recover in the regular medium for another three days (C). The morphological evaluations were done by a microscopy and representative image was shown (magnification 10×10). Bar = 100 μm.

Cellular viability was analyzed by MTT assay. As shown in **Fig 1A**, Doc inhibited the cellular viability of SKOV3 and A2780 cells in a dose-dependent manner from 0.05 to 100 nM. Surprisingly, Doc did not further reduce cell viability at concentrations higher than 100 nM **(Fig 1A)**. Microscopic imaging analysis was performed to observe the growth of cells. Compared with the control group, it seemed that Doc inhibited the growth of SKOV3 and A2780 cells, but there were not significant differences between different concentrations of Doc (**Fig 1B and 1C**). Although some cells were killed, a lot of cells were still alive and underwent morphological change, especially when treatment with Doc at 800 nM (**Fig 1B and 1C**). We decided to use 800 nM Doc for future experiments, as mentioned in the experimental method.

### Doc induces the formation of PGCCs

**Fig 2:**
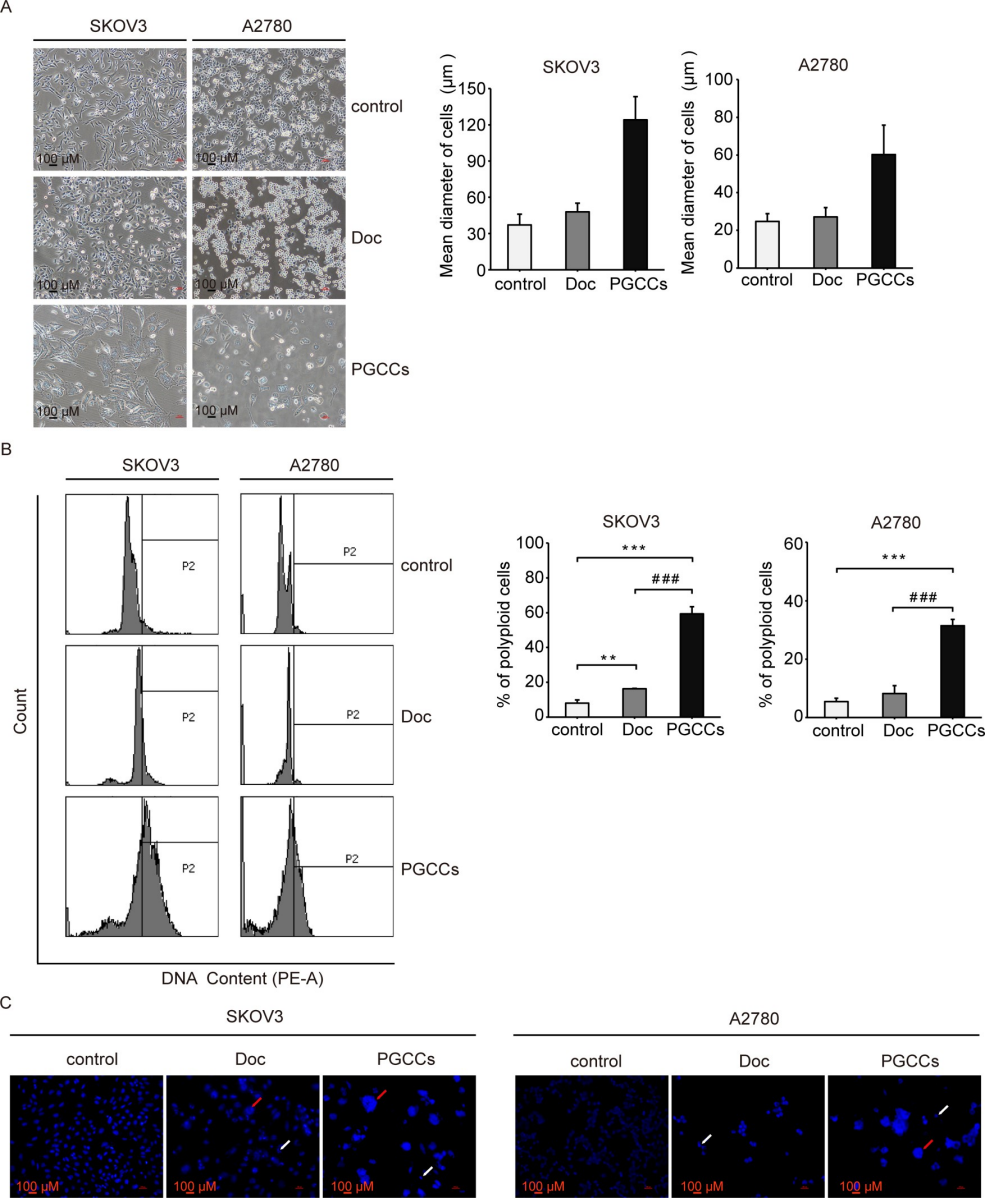
Doc promoted the formation of PGCCs. (A) The morphology of cells was imaged under a microscope followed by processing with the NIS-Elements D 3.2 software. Representative image was shown (magnification 10×10). Bar = 100 μm. Bar graphs showed the diameter of cells (n = 50). (B) Polyploid phenomena were analyzed by flow cytometry. Representative histogram was shown, and the region marked by black line indicated by P2 showed the polyploid cells (DNA>4N). Bar graphs showed the percentage of polyploid cells (n = 3). *P* values were calculated by One-way ANOVA. **P<0.01, ***P<0.001 (*vs*. control); ^###^*P*<0.001 (PGCCs *vs*. Doc). (C) Hochest 33342 staining was used to reveal the morphology of nucleus. Representative image was shown (magnification 10×20). Red arrow indicated multinuclear cell and white arrow indicated micronucleus cell. Bar = 100 μm.

We observed that most of viable cells exhibited considerable flattening and increase in surface area after Doc treatment (**Fig 2A**). The diameter of SKOV3 and A2780 cells was increased continuously from 37.2 ± 8.9 and 24.7 ± 4.1 μm in the control group to 124.1 ± 19.3 and 60.3 ± 15.5 μm in the PGCCs group, which was roughly threefold higher than the control group (**Fig 2A**). According to the criterion from the long-term experimental data and observation [14], most of cells in the PGGCs group could be designated as PGCCs. To identify PGCCs as polyploid cells, DNA content analysis was performed to reveal the change of PGCCs over time. As shown in **Fig 2B**, the percentage of polyploid cells (DNA>4N) was increased from 7.97 ± 1.92 and 5.47 ± 1.10% in the control group to 16.23 ± 0.32 and 8.23 ± 2.70% in the Doc group, and further to 59.33 ± 4.12 and 31.43 ± 2.23% in the PGCCs group. To confirm the existence of PGCCs after Doc treatment, the morphology of nucleus was further observed after Hoechst 33342 staining. Larger nuclei could be clearly seen under a fluorescence microscope after Doc treatment, especially in the PGCCs group (**Fig 2C**). Meanwhile, some cells had multiple nuclei and a small number of cells possessed micronuclei (**Fig 2C**). These results suggested that Doc induced the formation of PGCCs in SKOV3 and A2780 cells, and the chromosomal structure of PGCCs became abnormal, resulting in the production of multinucleus and micronucleus cells.

### Doc induces G2/M cell cycle arrest and inhibits proliferation

**Fig 3:**
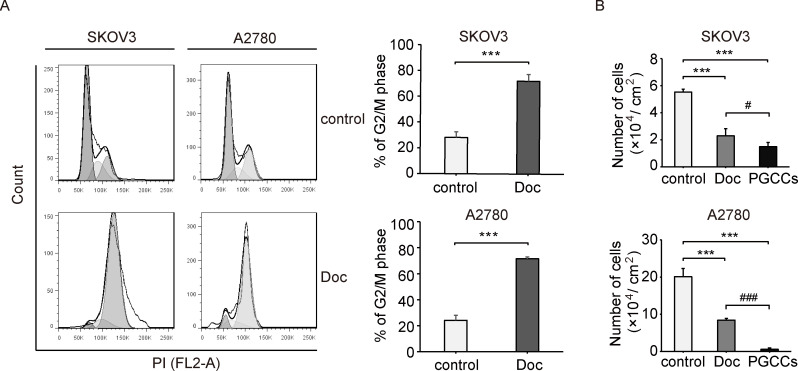
Doc induced G2/M cell cycle arrest and inhibited proliferation. (A) The cell cycle distribution was analyzed by flow cytometry followed by processing with the Flow Jo 7.6 software. Representative histogram was shown. Bar graphs showed the percentage of cells in G2/M phase (n = 3). *P* values were calculated using the student’s t test. ***P<0.001 (*vs*. control). (B) The number of cells was counted and bar graphs showed the cell density (n = 3). *P* values were calculated using the One-way ANOVA. ***P<0.001 (*vs*. control); ^#^*P*<0.05, ^###^*P*<0.001 (PGCCs *vs*. Doc).

To determine the effect of Doc on cell cycle progression, the cell cycle distribution was evaluated by flow cytometry. It seemed that Doc induced G2/M cell cycle arrest in SKOV3 and A2780 cells (**Fig 3A)**. As shown in **Fig 3A**, the percentage of G2/M phase was significantly increased from 24.12 ± 3.88 and 28.16 ± 4.42% in the control group to 71.48 ± 1.50 and 72.06 ± 5.21% in the Doc group. Cell cycle arrest can mediate the reduction of cell numbers [15]. As shown in **Fig 3B,** the number of cells was reduced from 5.53 ± 0.20 and 20.09 ± 2.22 (× 10^4^ cells /cm^2^) to 2.30 ± 0.54 and 8.40 ± 0.48 (× 10^4^ cells /cm^2^) in the Doc group. Moreover, the number of cells was further reduced to 1.50 ± 0.30 and 0.83 ± 0.22 (× 10^4^ cells /cm^2^) in the PGCCs group, significantly less than that in the control group and Doc group (**Fig 3B**). These results suggested that Doc induced G2/M cell cycle arrest and inhibited the proliferation in SKOV3 and A2780 cells.

### Doc activates persistent DDR signaling

**Fig 4:**
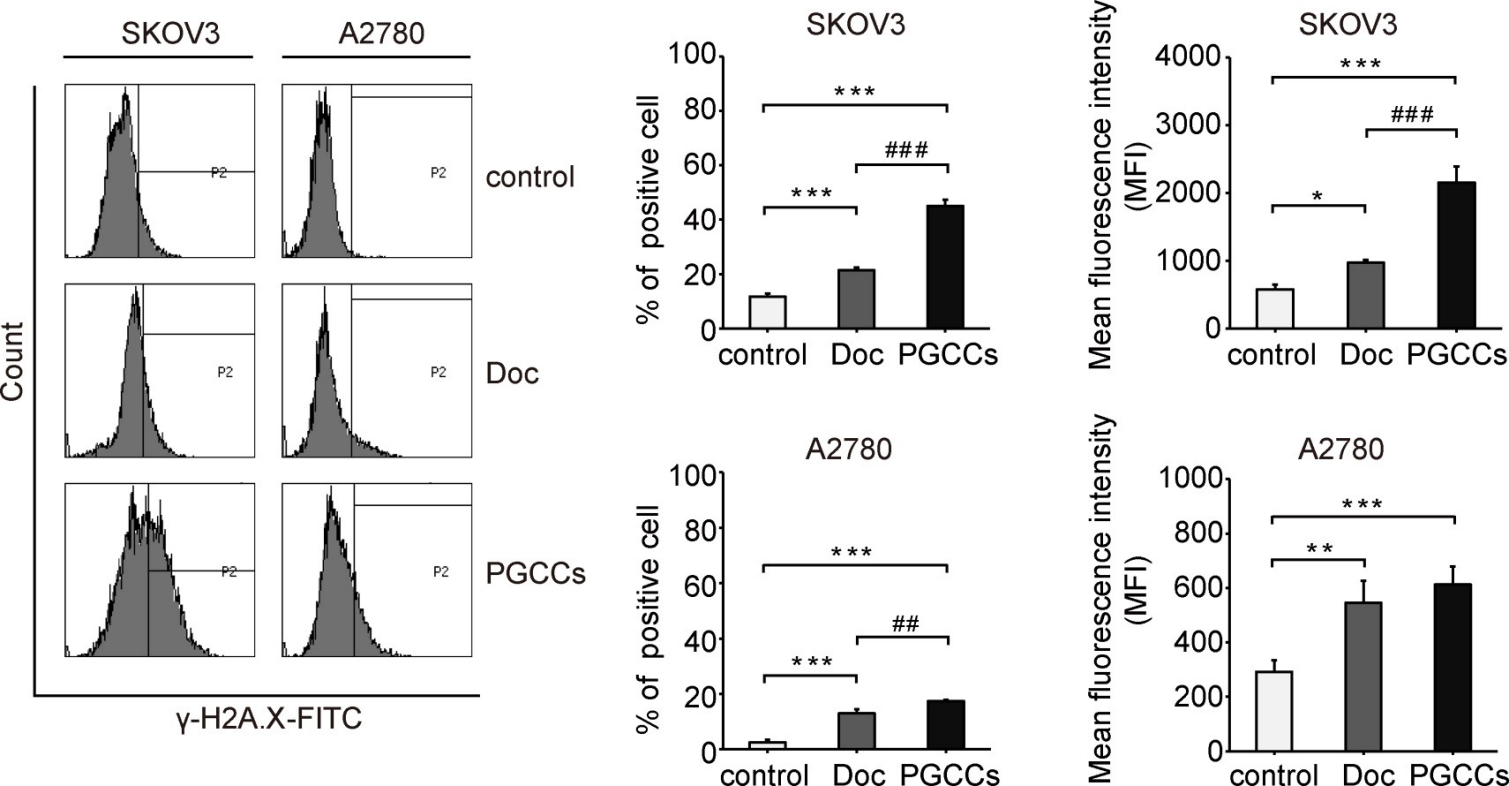
Doc activated persistent DDR signaling. The expression of γ-H2A.X was detected by flow cytometry followed by processing with the BD FACSDiva software. Representative histogram was shown, and the region marked by the black line indicated by P2 showed the γ-H2A.X-positive cells. Bar graphs showed the percentage of γ-H2A.X-positive cells (left, n = 3) and the mean fluorescence intensity (MFI) of γ-H2A.X (right, n = 3). *P* values were calculated by One-way ANOVA. *P<0.05, **P<0.01, ***P<0.001 (*vs*. control); ^##^*P*<0.01, ^###^*P*<0.001 (PGCCs *vs*. Doc).

Doc is a cytotoxic agent that usually leads to DNA double-stranded breaks (DSBs) [16]. Subsequently, γ-H2A.X, an early marker of DSBs, guides the recruitment of DDR factors to trigger DDR signaling [17]. After Doc treatment, DDR signaling was evaluated by flow cytometry. The expression of γ-H2A.X was increased over time in SKOV3 and A2780 cells (**Fig 4)**. As shown in **Fig 4,** the percentage of γ-H2A.X-positive cells was increased from 11.7 ± 1.1 and 2.6 ± 0.9% in the control group to 21.5 ± 1.0 and 13.0 ± 1.5% in the Doc group, and further to 45.0 ± 2.4 and 17.4 ± 2.4% in the PGCCs group. The mean fluorescence intensity (MFI) of γ-H2A.X was increased from (579 ± 70) and (292 ± 42) in the control group to (971 ± 42) and (546 ± 80) in the Doc group, and further to (2154 ± 241) and (613 ± 65) in the PGCCs group (**Fig 4**). These results suggested that Doc activated persistent DDR signaling with the formation of PGCCs in SKOV3 and A2780 cells.

### β-galactosidase activity increases and mitochondrial membrane potential loses in PGCCs

**Fig 5:**
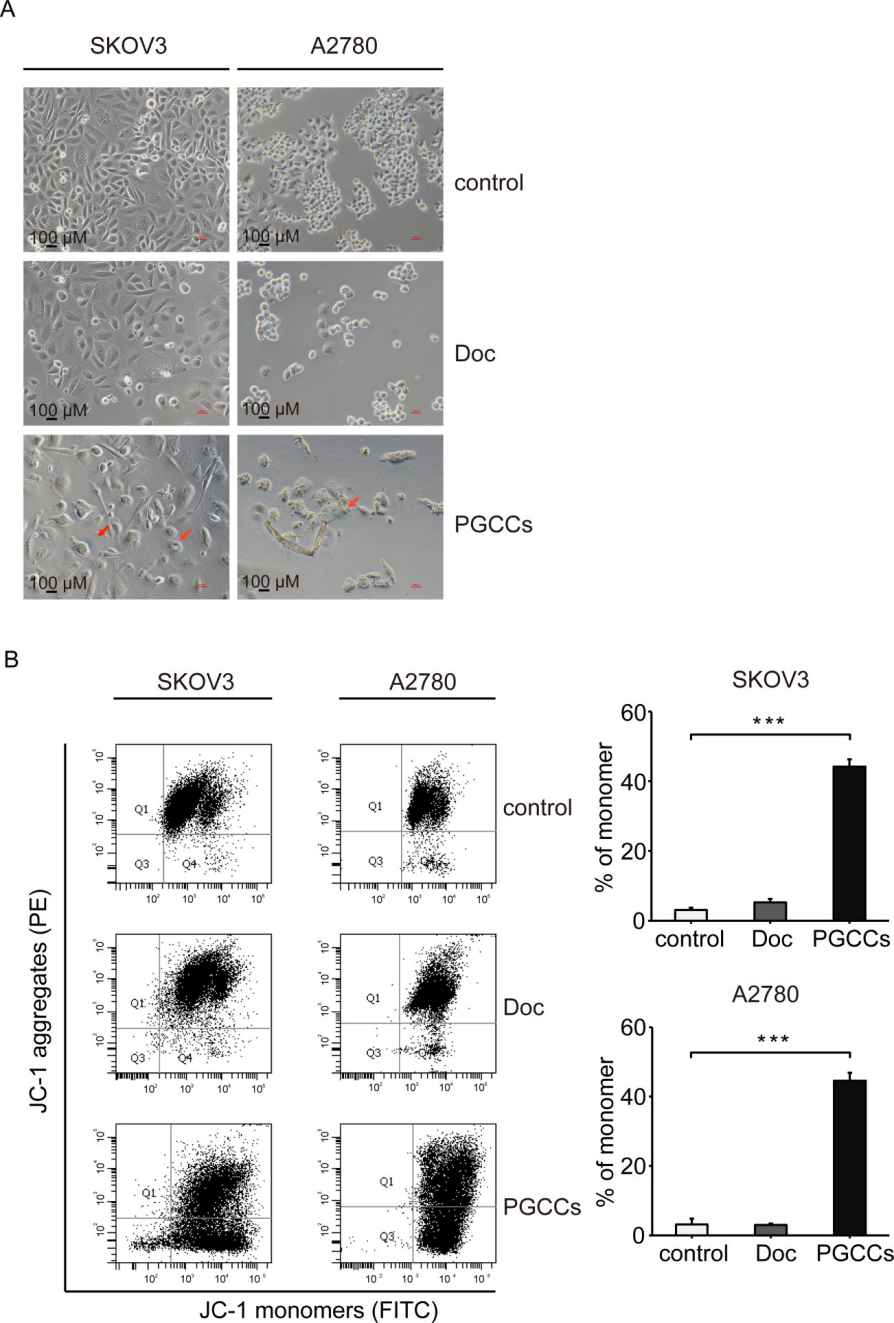
The SA-β-Gal activity was increased and the mitochondrial membrane potential was lost in PGCCs. (A) SA-β-Gal staining to reveal the activity of β-galactosidase. Representative image was shown (magnification 10×20). Black arrows indicated the SA-β-Gal-positive cell. Bar = 100 μm. (B) JC-1 staining to reveal the mitochondrial membrane potential. Representative scatter plot was shown. Bar graphs showed the percentage of JC-1 monomeric form (n = 3). *P* values were calculated by One-way ANOVA. ***P<0.001 (*vs*. control).

A primary characteristic of senescent cells is the increased activity of lysosomal β-galactosidase, which is exploited as senescence-associated β-galactosidase (SA-β-Gal) [18]. To confirm whether Doc induce cellular senescence, SA-β-Gal assay was performed in SKOV3 and A2780 cells. The activity of β-galactosidase was rather low in the control group and Doc group (**Fig 5A**). However, the level of SA-β-Gal was increased in the PGCCs group (**Fig 5A**). These results suggested that Doc activates SA-β-Gal activity with the formation of PGCCs. To determine the effect of Doc on mitochondrial function, we further monitored the changes in mitochondrial membrane potential by flow cytometry after staining with JC-1 in SKOV3 and A2780 cells. JC-1 existed in the form of aggregates and almost no monomeric form could be detected in the control group and Doc group (**Fig 5B**). However, the proportion of JC-1 monomeric form was significantly increased from 3.1 ± 1.7 and 3.1 ± 0.6% in the control group to 44.6 ± 2.3% and 44.2 ± 2.1% in the PGCCs group (**Fig 5B**). The results suggested that Doc reduced mitochondrial membrane potential with the formation of PGCCs.

### The expression of stem cell markers was upregulated in PGCCs

**Fig 6:**
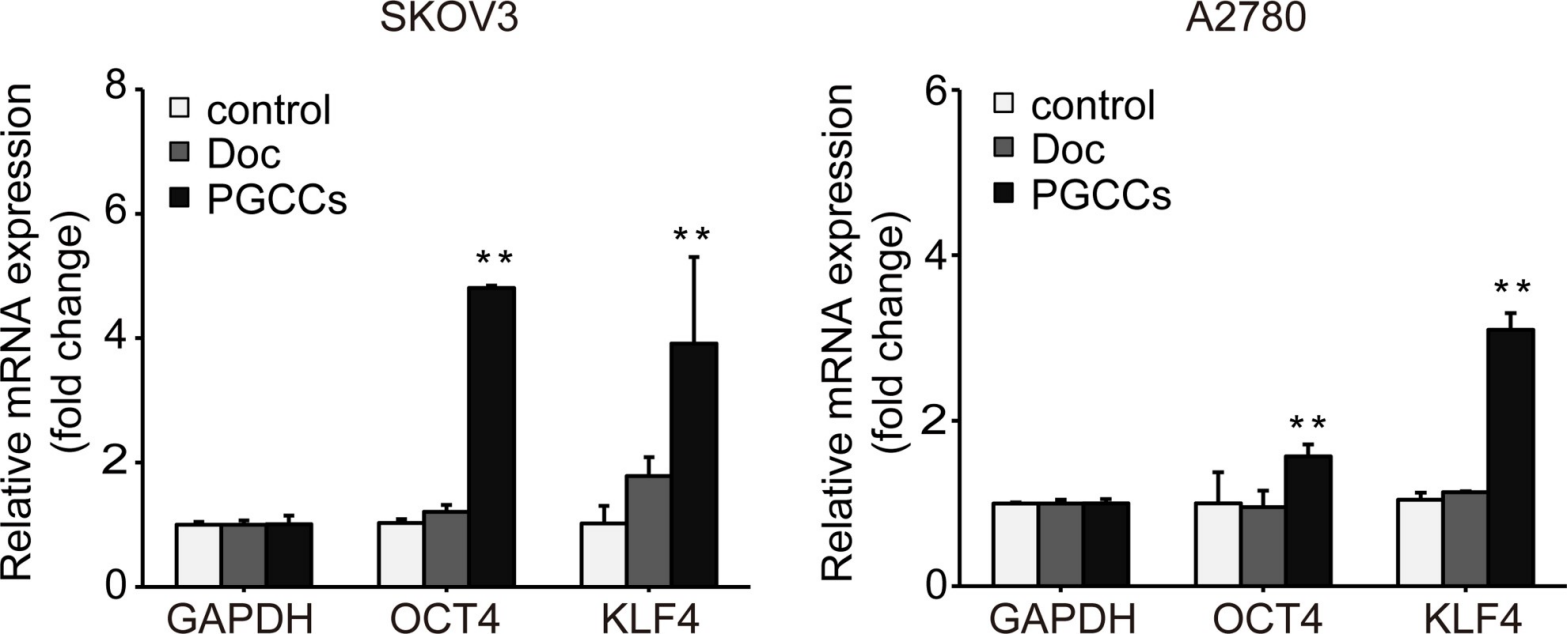
The expression of stem cell markers was upregulated in PGCCs. Relative mRNA level of *KLF4* and *OCT4* was determined by RT-qPCR. The values were normalized to GAPDH and relative to the control group on the basis of 2^-ΔΔCt^ method. *P* values were calculated using the student’s t test. **P<0.01 (*vs*. control).

PGCCs have been confirmed to have cancer stem cell (CSC)-like properties [19]. We focused on the expression of transcription factors including OCT4 and KLF4, which are stem cell markers [20]. After Doc treatment, the mRNA level of *OCT4* and *KLF4* was analyzed by RT-qPCR in SKOV3 and A2780 cells. Compared with the control group, the mRNA level of *OCT4* was not significantly increased in the Doc group, but increased 4.81 ± 0.04 and 1.57 ± 0.14-fold in the PGCCs group (**Fig 6)**. Compared with the control group, the mRNA level of *KLF4* was not also significantly increased in the Doc group, but increased 3.91 ± 1.39 and 3.10 ± 0.20-fold in the PGCCs group (**Fig 6**). These results suggested that the expression of stem cell markers was upregulated in PGCCs.

## Discussion

Among the available chemotherapy drugs, Doc is the standard of care, either alone or in combination with other drugs [21]. Chemotherapy drugs can eliminate tumors, but most tumors eventually relapse and become drug resistant. Some cancer cells exposed to Doc escape its cytotoxic effect and are characterized by polyploidization, generating PGCCs [22, 23]. Although most of PGCCs succumb to cell death, some can survive and acquire cancer stem cells properties [19, 23]. PGCCs are also considered to be a key factor in the process of tumorigenesis, metastasis and therapy resistance [24, 25]. It seems necessary to pay more attention to PGCCs, a cancer cell subpopulation, in different aspects of tumor biology and therapy. Therefore, a simple model system is needed to study these cells in detail. In this study, SKOV3 and A2780 cells were treated with Doc to induce the formation of PGCCs. On the basis of this model, we explored the formation mechanism of PGCCs and observed their biological behavior.

In our experiment, Doc induced G2/M cell cycle arrest followed by the generation of PGCCs. PGCCs can be generated by cell cycle failure, such as endoreplication, and the failure in mitosis is one of the main mechanisms for switching on an endoreplication cell cycle [26]. Paclitaxel-treated cells have defects in mitotic spindle assembly, chromosome segregation and cell division, which can induce surviving cells to enter an endoreplication cell cycle to form PGCCs [27]. The effector mechanism of Doc may be similar to that of paclitaxel, which stabilizes the microtubule polymer and blocks the progression of mitosis to induce G2/M cell cycle arrest, ultimately driving cancer cells into an endoreplication cell cycle. Following endoreplication, cancer cells can arrest the mitotic cell cycle and enter the giant cell cycle to form PGCCs [27, 28], which allow cells to survive during mitotic catastrophe or genotoxic stress.

Chemotherapy can induce DNA damage to trigger the DNA repair mechanism, for which can activate the cell cycle checkpoints and arrest cell cycle to repair the damage [29]. Because the DNA damage is severe or irreparable, Doc usually leads to DSB, for which DDR signaling remains persistently activated [30]. Both cellular polyploidization and senescence are induced by an irreparable DSB followed by activation of persistent DDR signaling [31]. Our study also suggested that persistent DDR signaling was activated with the formation of PGCCs and senescence in SKOV3 and A2780 cells. Aberrant activation of DDR signaling is strongly correlated with resistance of cancer cells to genotoxic anti-tumor therapeutics [32, 33]. Many reports associate increased DDR signaling with cancer cell survival after chemotherapy, making this pathway an attractive target for overcoming cancer chemoresistance.

Cellular senescence is a multi-functional cell fate characterized by the arrest of proliferation while maintaining metabolic activity and viability [34]. Initially, senescent cells received little attention in the cancer research because they were traditionally thought to lack the ability to proliferate. More recently, cellular senescence has been suggested to contribute to tumor recurrence, invasiveness and metastasis [35, 36]. The relationship between PGCCs and senescent cells is still under debate. PGCCs are traditionally considered to be senescent and tumor suppressive, as they are described as non-dividing, flattened tumor cells that are irreversibly arrested in either the G0/G1 or G2/M state and express β-galactosidase activity [37]. On the contrary, PGCCs have also been characterized as non-senescent, as Zhang et al. found no positive staining in β-galactosidase staining of PGCCs formed by HEY, SKOV3 and MDA-MB-231 [19]. Furthermore, PGCCs have been described as "pseudo-senescent" and may escape from senescence by forming proliferative aneuploidy progeny [38]. In our study, PGCCs exhibited many characteristics of senescence, including cell hypertrophy and flattening, irreversible arrest of cell cycle progression and proliferation, increased β-galactosidase activity, and mitochondrial dysfunction. Many of the phenotypic changes associated with the senescent program are relevant to understanding the pathophysiological functions of senescent cells.

Mitochondrial membrane potential is critical for maintaining the physiological function of mitochondrial metabolism [39]. In our study, loss of mitochondrial membrane potential was observed with the generation of PGCCs and senescence. Kaplon and colleagues argued that senescent cells must reprogram their metabolism to support their metabolic demands [40]. Senescent cells exhibit metabolic changes such as increases in glycolysis, mitochondrial metabolism, and autophagy [8], which are extremely complex. Different metabolic signatures and changes lead to different responses to chemotherapeutic drugs. Drug resistance and chemosensitivity of cancer cells depend on the metabolic type of the mitochondria. The chemosensitive cells such as A2780 cells show a glycolytic phenotype, whereas the resistant cells show a switch to oxidative phosphorylation [41]. In further research, more attention should be paid to the metabolic changes of mitochondria to explain the resistance to chemotherapy.

Although the role of stem cell factors in PGCCs and senescent cells remains unclear, their increased expression is frequently observed in various types of tumors and is associated with cancer progression, resistance to therapy, and poor prognosis [42, 43]. Initially, researchers found that PGCCs are not only alive, but can also divide asymmetrically and give rise to progeny cancer cells with cancer stem-like properties [44]. Min Thura and colleagues demonstrated that PRL3-induced PGCCs, like other previously described PGCCs, also possess stem cell-like properties [45]. TIS could alter the stem cell-like properties of malignant cells, which exert their deleterious, highly aggressive growth potential upon escape from cell cycle blockade and are enriched in recurrent tumors [46]. There is reason to believe that PGCCs and senescent cells with stem cell-like properties are the key for tumor recurrence.

## Supporting information

S1 File The raw for MTT in Fig 1. (XLSX)

S2 File The raw for diamerer and percentage of polyploidy cells in Fig 2. (XLS)

S3 File The raw for cell cycle in Fig 3. (XLS)

S4 File The raw for number of cells in Fig 3. (XLSX)

S5 File The raw for percentage of positive cell in Fig 4. (XLSX)

S6 File The raw for MFI in Fig 4. (XLSX)

S7 File The raw for JC-1 monomers in Fig 5. (XLSX)

S8 File The raw for qPCR in Fig 6. (XLSX)
